# Human IAPP is a contributor to painful diabetic peripheral neuropathy

**DOI:** 10.1172/JCI156993

**Published:** 2023-04-17

**Authors:** Mohammed M.H. Albariqi, Sabine Versteeg, Elisabeth M. Brakkee, J. Henk Coert, Barend O.W. Elenbaas, Judith Prado, C. Erik Hack, Jo W.M. Höppener, Niels Eijkelkamp

**Affiliations:** 1 Center for Translational Immunology, University Medical Center Utrecht, Utrecht University, Utrecht, Netherlands.; 2 Institute of Applied Genomic Technologies, Health Sector, King Abdulaziz City for Science and Technology, Riyadh, Saudi Arabia.; 3 Department of Plastic and Reconstructive Surgery, University Medical Center Utrecht, Utrecht University, Utrecht, Netherlands.; 4 Membrane Biochemistry and Biophysics, Bijvoet Centre for Biomolecular Research, and; 5 Center for Molecular Medicine, University Medical Center Utrecht, Utrecht University, Utrecht, Netherlands.

**Keywords:** Immunology, Neuroscience, Diabetes, Pain, Protein misfolding

## Abstract

Peripheral neuropathy is a frequent complication of type 2 diabetes mellitus (T2DM). We investigated whether human islet amyloid polypeptide (hIAPP), which forms pathogenic aggregates that damage pancreatic islet β cells in T2DM, is involved in T2DM-associated peripheral neuropathy. In vitro, hIAPP incubation with sensory neurons reduced neurite outgrowth and increased levels of mitochondrial reactive oxygen species. hIAPP-transgenic mice, which have elevated plasma hIAPP levels without hyperglycemia, developed peripheral neuropathy as evidenced by pain-associated behavior and reduced intraepidermal nerve fiber (IENF) density. Similarly, hIAPP Ob/Ob mice, which have hyperglycemia in combination with elevated plasma hIAPP levels, had signs of neuropathy, although more aggravated. In wild-type mice, intraplantar and intravenous hIAPP injections induced long-lasting allodynia and decreased IENF density. Non-aggregating murine IAPP, mutated hIAPP (pramlintide), or hIAPP with pharmacologically inhibited aggregation did not induce these effects. T2DM patients had reduced IENF density and more hIAPP oligomers in the skin compared with non-T2DM controls. Thus, we provide evidence that hIAPP aggregation is neurotoxic and mediates peripheral neuropathy in mice. The increased abundance of hIAPP aggregates in the skin of T2DM patients supports the notion that hIAPP is a potential contributor to T2DM neuropathy in humans.

## Graphical abstract

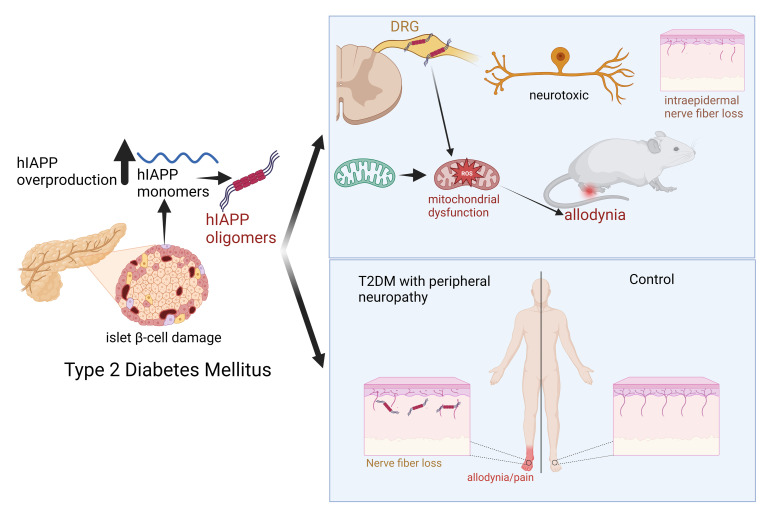

## Introduction

Diabetes mellitus (DM) is a metabolic disorder that affects around 463 million individuals globally (1). DM is one of the most significant worldwide health issues, diminishing quality of life and increasing morbidity and mortality (2). Type 2 DM (T2DM) is the most common type of DM, characterized by insulin resistance and β cell dysfunction (3). Diabetic peripheral neuropathy (DPN), a debilitating complication of DM, affects approximately 50% of T2DM patients (4). The pathogenesis of this complication is not fully understood. Control of hyperglycemia is not sufficient to attenuate DPN. Moreover, neuropathy can be present already in the prediabetic state, when hyperglycemia has not yet developed. Thus, apparently hyperglycemia is not the only cause of DPN (5).

Pancreatic islet amyloid is a characteristic histopathological feature of T2DM, found in approximately 90% of T2DM patients. Human islet amyloid polypeptide (hIAPP) is the main component of these amyloid deposits. IAPP, or amylin, is a 37–amino acid polypeptide hormone belonging to the calcitonin gene–related peptide (CGRP) family. It is co-secreted with insulin by pancreatic islet β cells. As a monomer, hIAPP is a soluble protein that plays a role in glucose regulation by enhancing satiety, reducing gastric emptying, and suppressing glucagon release (6). Islet amyloid formation has been associated with β cell failure in humans, monkeys, and cats with T2DM (6, 7). A hallmark of amyloid fibrils is their distinctive cross β structure, which is created by the sequential stacking of β sheets from fibril-forming protein molecules (8). Pre-fibrillar oligomer formation during protein aggregation in T2DM has been associated with cellular toxicity and a reduction in organ and cell function (9).

In T2DM, hIAPP is produced in large quantities. At high concentrations hIAPP forms toxic aggregates (oligomers and amyloid fibrils), causing β cell death and possibly damage in other tissues (10). In contrast, IAPP from mice and rats does not form amyloid, because of a different amino acid sequence that prevents formation of toxic aggregates, and these rodents do not “spontaneously” develop T2DM (11). hIAPP amyloid deposits are not exclusively found in the pancreatic islets of T2DM patients, but are also found in other organs/tissues such as brain, heart, and kidney (12).

Considering that peripheral neuropathy is a common complication of amyloid diseases, such as familial amyloid polyneuropathy (12), that T2DM is an amyloid disease (6, 12), and that hIAPP aggregation is not restricted to the pancreas, we investigated whether hIAPP is involved in the development of diabetic peripheral neuropathy.

## Results

To date, most research into T2DM neuropathy involves rodent models with metabolic disturbance, e.g., mice with a leptin deficiency (Ob/Ob mice). However, these models are not ideal, because they lack elevated amyloidogenic IAPP and β cell death in the pancreas as occurs in patients with T2DM when disease progresses (13).

**Figure 1:**
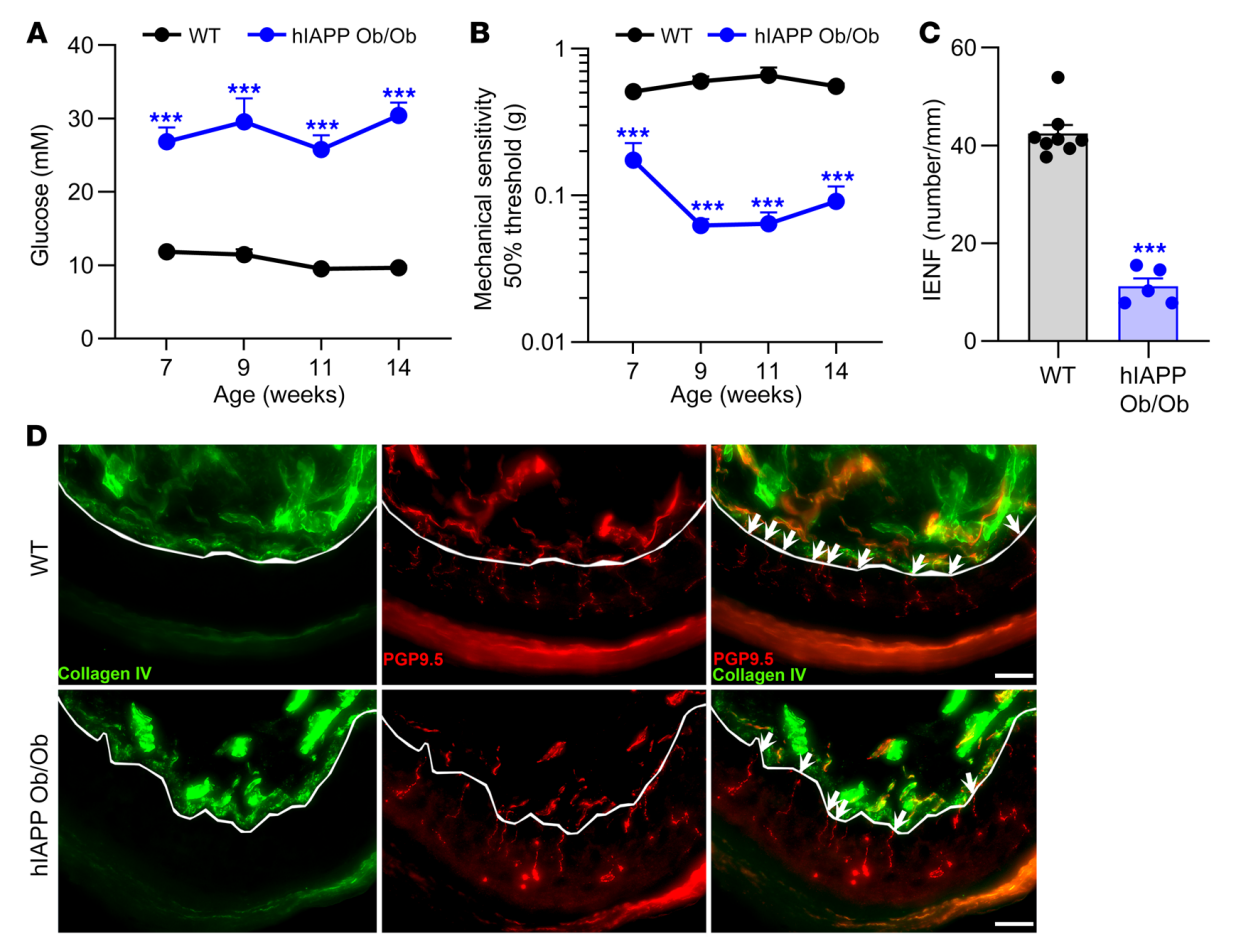
hIAPP Ob/Ob mice have features of T2DM neuropathy. (**A**) Nonfasting blood glucose levels in WT (*n* = 8) and hIAPP Ob/Ob (*n* = 5–8) mice; 2-way ANOVA with Šidák’s test, ***P < 0.001. (**B**) Mechanical threshold of the plantar surface of WT (*n* = 8) and hIAPP Ob/Ob (*n* = 7) mice; 2-way ANOVA with Šidák’s test, ***P < 0.001. (**C**) Number of nerve fibers crossing from dermis to epidermis in WT (*n* = 8) and hIAPP Ob/Ob (*n* = 5) mice at age 15 weeks; unpaired *t* test, ***P < 0.001. (**D**) Representative images of paw skin of WT and hIAPP Ob/Ob mice stained for the pan-neuronal marker PGP9.5 and collagen IV (lines indicate the border between dermis and epidermis; white arrows represent IENF; scale bars: 20 μm). All experiments were performed in male mice. Data are expressed as mean ± SEM.

Therefore, we evaluated whether signs of T2DM neuropathy develop in a mouse model of T2DM in which hIAPP is expressed by the β cells of pancreatic islets (hIAPP Ob/Ob). hIAPP Ob/Ob mice had significantly elevated nonfasting (Figure 1A) and fasting (14) blood glucose levels, indicating they were diabetic. To assess development of allodynia, we used the Von Frey test, which measures the withdrawal response to innocuous mechanical stimulation. hIAPP Ob/Ob mice had allodynia from 7 weeks of age as compared with WT mice (Figure 1B). Moreover, at 15 weeks of age, hIAPP Ob/Ob mice had reduced intraepidermal nerve fiber (IENF) density in the plantar skin compared with WT mice (Figure 1, C and D). Thus, hIAPP Ob/Ob mice not only have metabolic characteristics of T2DM, but also show signs of diabetic neuropathy.

**Figure 2:**
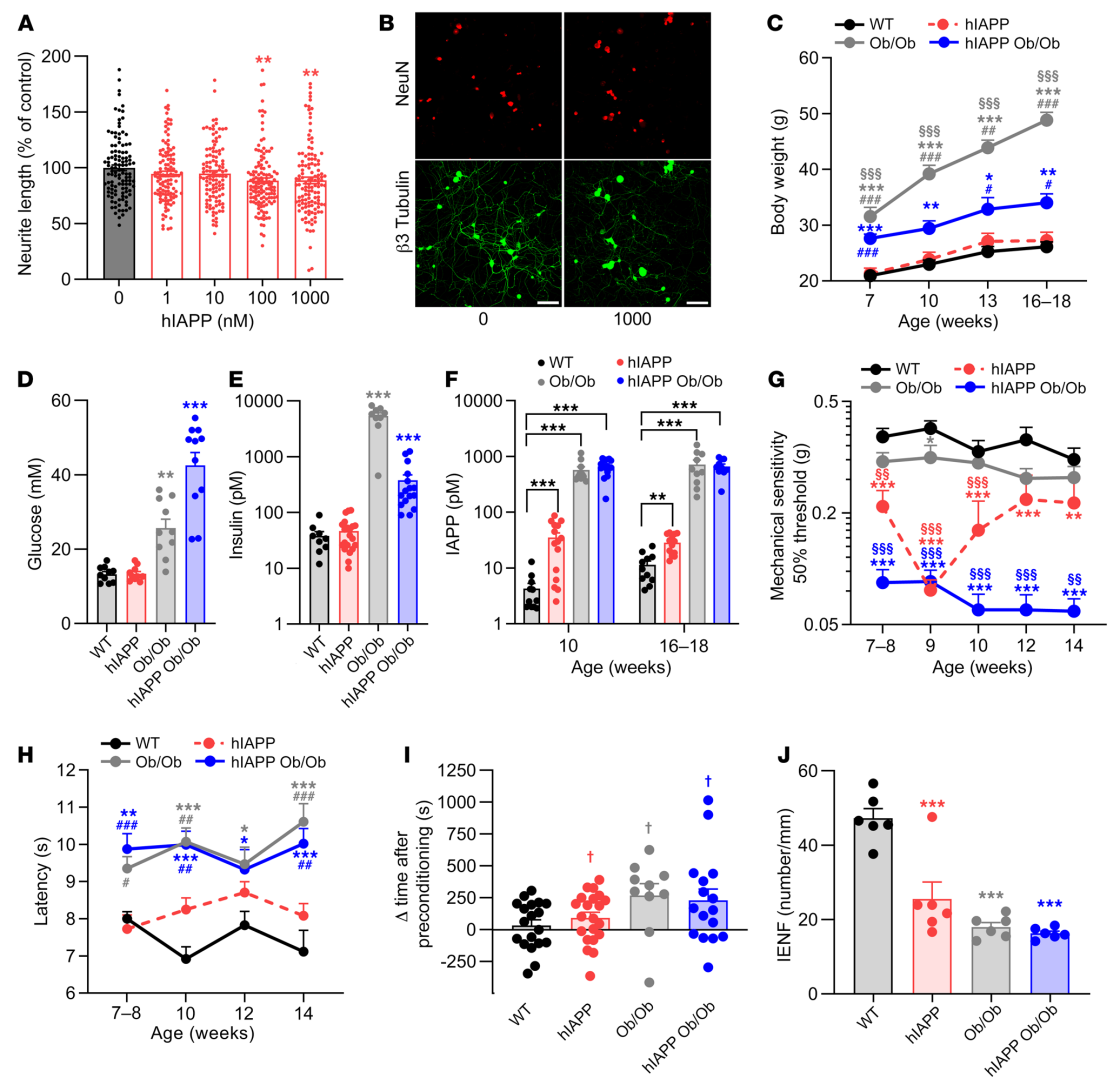
Human IAPP is neurotoxic to sensory neurons and hIAPP mice develop signs of neuropathy, also in the absence of hyperglycemia. (**A**) Sensory neurons were treated for 24 hours with different concentrations of hIAPP. The average neurite length per neuron was assessed and expressed as the percentage of the average neurite length per neuron of vehicle-treated neurons (*n* = 8; *n* represents a DRG culture of 1 mouse; male and female mice were used). (**B**) Representative images of DRGs stained for β_3_-tubulin (green) and NeuN (red), indicating the neurite outgrowth (green) and the soma number (red) (scale bars: 50 μm). (**C**) Body weight of WT (*n* = 12), hIAPP (*n* = 15), Ob/Ob (*n* = 10), and hIAPP Ob/Ob (*n* = 15) mice. (**D**–**F**) Nonfasting plasma glucose level (**D**), nonfasting plasma insulin level (**E**) (mice 16–18 weeks old in **D** and **E**), and nonfasting plasma IAPP levels (**F**) in WT, hIAPP, Ob/Ob, and hIAPP Ob/Ob mice. (**G**) Mechanical threshold of WT (*n* = 11), hIAPP (*n* = 23), Ob/Ob (*n* = 21), and hIAPP Ob/Ob (*n* = 14) mice. (**H**) Thermal sensitivity of WT (*n* = 11), hIAPP (*n* = 15), Ob/Ob (*n* = 12), and hIAPP Ob/Ob (*n* = 10) mice. (**I**) Conditioned place preference to reveal the presence of non-evoked pain. Mice were conditioned with gabapentin for 3 consecutive days and time spent in the conditioning compartment between the post- and preconditioning phases at 15–17 weeks of age. (**J**) Quantification of IENFs of the hind paw of mice at 16–18 weeks of age. All experiments were performed with male and female mice. (**A**, **D**, and **E**) One-way ANOVA with Dunnett’s test; **P < 0.01, ***P < 0.001. (**C**, **F**–**H**, and **J**) Two-way ANOVA with Tukey’s test; *P < 0.05, **P < 0.01, ***P < 0.001 vs. WT; ^§§^*P* < 0.01, ^§§§^*P* < 0.001 vs. Ob/Ob; ^#^*P* < 0.05, ^##^*P* < 0.01, ^###^*P* < 0.001 vs. hIAPP. (**I**) One-sample *t* test; ^†^*P* < 0.05 post- vs. preconditioning. Data are expressed as mean ± SEM.

Next we assessed whether hIAPP alone, thus independently of hyperglycemia and/or obesity, is sufficient to induce signs of neuropathy. To that end, we first investigated whether hIAPP is neurotoxic by treating cultured mouse sensory neurons with hIAPP for 24 hours. Neurite outgrowth was reduced by 12% at concentrations of 100 and 1,000 nM hIAPP compared with vehicle (Figure 2, A and B), without inducing cell death (Supplemental Figure 1A; supplemental material available online with this article; https://doi.org/10.1172/JCI156993DS1).

Next we measured, over a time course of 7–18 weeks of age, T2DM-associated parameters and pain-associated behaviors in hIAPP mice. hIAPP mice had body weight and plasma glucose and plasma insulin levels comparable to those of WT mice (Figure 2, C–E). In contrast, plasma IAPP levels were increased compared with those of WT mice (Figure 2F). The body weight of male hIAPP and WT mice was higher than that of female mice (Supplemental Figure 1B). Male and female mice did not differ in other of these parameters (Supplemental Figure 1, C–F). Thus, hIAPP mice do not have DM and provide a model to study the effects of hIAPP independently of hyperglycemia and obesity. Intriguingly, hIAPP mice had lower mechanical thresholds as compared with WT mice (Figure 2G). In female hIAPP mice mechanical thresholds were lower than in males (Supplemental Figure 2A). In contrast, thermal sensitivity in male and female hIAPP mice did not differ from that in WT mice (Figure 2H). To further assess pain-associated behavior, mice approximately 4 months of age were subjected to a conditioned place preference test with the neuropathic painkiller gabapentin (15), as a measure of non-evoked pain. Male and female hIAPP mice, but not WT mice, showed place preference after conditioning with gabapentin compared with preconditioning (Figure 2I). Overall, these data indicate that hIAPP expression is sufficient to induce pain-associated behaviors in non-obese and non-diabetic mice.

To assess whether hIAPP mice differ in their development of neuropathy compared with the more commonly used Ob/Ob mouse models, we compared hIAPP mice with obese mice (Ob/Ob), and hIAPP Ob/Ob mice. Notably, Ob/Ob and hIAPP Ob/Ob mice had increased body weight, plasma insulin levels, and nonfasted plasma glucose levels (Figure 2, C–E), in line with earlier reports of elevated fasted blood glucose levels in these mice (14, 16). hIAPP Ob/Ob mice gained less body weight compared with Ob/Ob mice because hIAPP Ob/Ob mice have more severe DM (14), which is associated with weight loss (17). Ob/Ob mice also showed significant mechanical hypersensitivity at 9 weeks of age, but this was less severe than in hIAPP mice or hIAPP Ob/Ob mice. While hIAPP mice did not have deficits in thermal sensitivity, male and female Ob/Ob and hIAPP Ob/Ob mice had an increased latency to heat stimulation as compared with WT or hIAPP mice (Figure 2H). Male and female mice did not differ in these parameters (Supplemental Figure 2B). Ob/Ob and hIAPP Ob/Ob mice showed place preference after conditioning with gabapentin compared with preconditioning (Figure 2I).

Peripheral neuropathy may lead to loss of IENFs. Interestingly, the number of IENFs was reduced in male and female hIAPP mice, hIAPP Ob/Ob mice, and Ob/Ob mice compared with WT mice (Figure 2J). These parameters were comparable for male and female mice (Supplemental Figure 2C). Overall, these data indicate that hIAPP, in the absence of hyperglycemia and obesity, is sufficient to induce signs of peripheral neuropathy, and this neuropathy is more severe in diabetic and obese hIAPP mice (hIAPP Ob/Ob).

**Figure 3:**
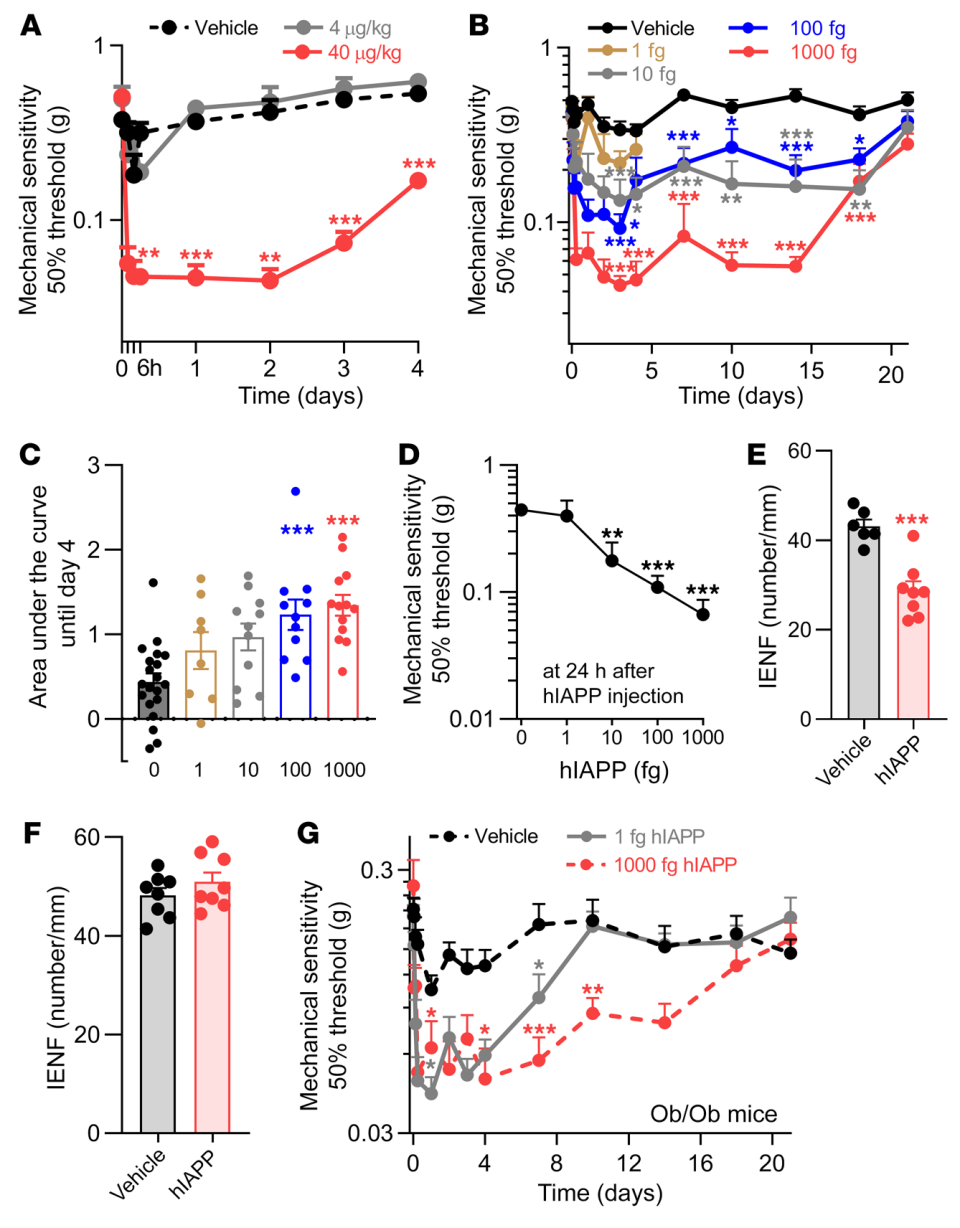
Human IAPP reduces mechanical thresholds and IENF density in WT mice. (**A** and **B**) Mechanical sensitivity of the hind paw after intravenous (40 μg/kg, *n* = 7; 4 μg/kg, *n* = 3; saline, *n* = 6) (**A**) or intraplantar injection of hIAPP (1 fg, *n* = 8; 10 fg, *n* = 11; 100 fg, *n* = 11; 1,000 fg, *n* = 13; saline, *n* = 20) (**B**). (**C**) Area under the curve of the reduction in mechanical threshold from day 0 to day 4 after intraplantar hIAPP injection of data shown in **B**. (**D**) Dose-response curve of hIAPP-induced mechanical allodynia measured at 1 day after injection. (**E** and **F**) Quantification of plantar IENFs at 6 days after intraplantar injection of 1,000 fg hIAPP or saline (**E**), or at 27 days after hIAPP injection (1,000 fg) when hypersensitivity had resolved (*n* = 8) (**F**). (**G**) Mechanical sensitivity of the hind paw after intraplantar injection of hIAPP (1 fg, *n* = 17; 1,000 fg, *n* = 12) or saline (*n* = 17) into male and female Ob/Ob mice. (**A**, **B**, and **G**) Two-way ANOVA with Dunnett’s test; *P < 0.05, **P < 0.01, ***P < 0.001 vs. vehicle injection. (**C** and **D**) One-way ANOVA with Dunnett’s test; **P < 0.01, ***P < 0.001 vs. vehicle injection (0 fg hIAPP). (**E** and **F**) Unpaired *t* test; ***P < 0.001. Data are expressed as mean ± SEM.

Peripheral and central administration of IAPP affects the sensory nervous system in rodents and causes endothelial dysfunction, vessel wall disruption, and neurological deficits (18). To test whether hIAPP administration to WT mice is sufficient to induce pain-associated behaviors, hIAPP was injected i.v. in WT mice. A single i.v. hIAPP injection at 40 μg/kg, but not at 4 μg/kg, reduced mechanical thresholds within 2 hours of injection until 4 days after injection (Figure 3A). The hIAPP dose that induced allodynia (40 μg/kg) results in a calculated (see Methods for calculation) maximal plasma level of hIAPP that is approximately 1,000 times higher than in hIAPP-transgenic mice (up to 100 pM) and/or humans with T2DM (up to 42 pM) (19). hIAPP has a half-life of only a few minutes (20), indicating that a short but strong rise in systemic hIAPP concentration is sufficient to cause long-lasting changes in peripheral sensory neurons. Intravenous hIAPP injection did not affect thermal sensitivity (Supplemental Figure 3A).

Next, we tested whether local injection of hIAPP into the hind paw of WT mice induces local signs of neuropathy. Intraplantar injection of hIAPP dose-dependently induced allodynia (Figure 3, B–D). The maximum hIAPP dose (1,000 fg, calculated plasma level of ~50 pM; see Methods for calculation) that elicited long-lasting mechanical hypersensitivity was in the same concentration range as found in blood of hIAPP mice and T2DM patients (19). At this dose, hIAPP reduced mechanical thresholds for at least 2 weeks (Figure 3B and Supplemental Figure 3B), and this reduction resolved within 3 weeks (Supplemental Figure 3C). IENFs measured in the plantar skin are predominantly unmyelinated (21). Intraplantar injection of 1,000 fg hIAPP reduced the density of IENFs compared with vehicle injection (Figure 3E).

Importantly, 1 week after hIAPP-induced hypersensitivity had resolved (Supplemental Figure 3C), the plantar skin IENF density was indistinguishable from that of vehicle-injected mice (Figure 3F). Thus, nerve fibers recover concurrently with the resolution of hIAPP-induced mechanical hypersensitivity. Protein oligomers and larger aggregates may induce mechanical hypersensitivity through inducing inflammation. Intraplantar injection of hIAPP did not trigger expression of IL-6, IL-1β, and TNF mRNA at 6 and 24 hours or 6 days after injection. hIAPP injection did increase F4/80 mRNA expression (a marker for macrophages) after 1 week of injection, but not at the other time points (Supplemental Figure 3, D–F).

hIAPP Ob/Ob mice had stronger mechanical allodynia (Figure 2G), but also higher hIAPP levels (Figure 2F), than hIAPP or Ob/Ob mice. Thus, we questioned whether hyperglycemic Ob/Ob mice are more sensitive to hIAPP-induced allodynia as compared with WT mice. Ob/Ob mice received 1 fg hIAPP intraplantarly, a dose that did not induce allodynia in WT mice (Figure 3B). Intriguingly, this dose increased mechanical sensitivity in Ob/Ob mice for almost 7 days (Figure 3G). These data suggest that hyperglycemia and obesity aggravate hIAPP-induced allodynia.

**Figure 4:**
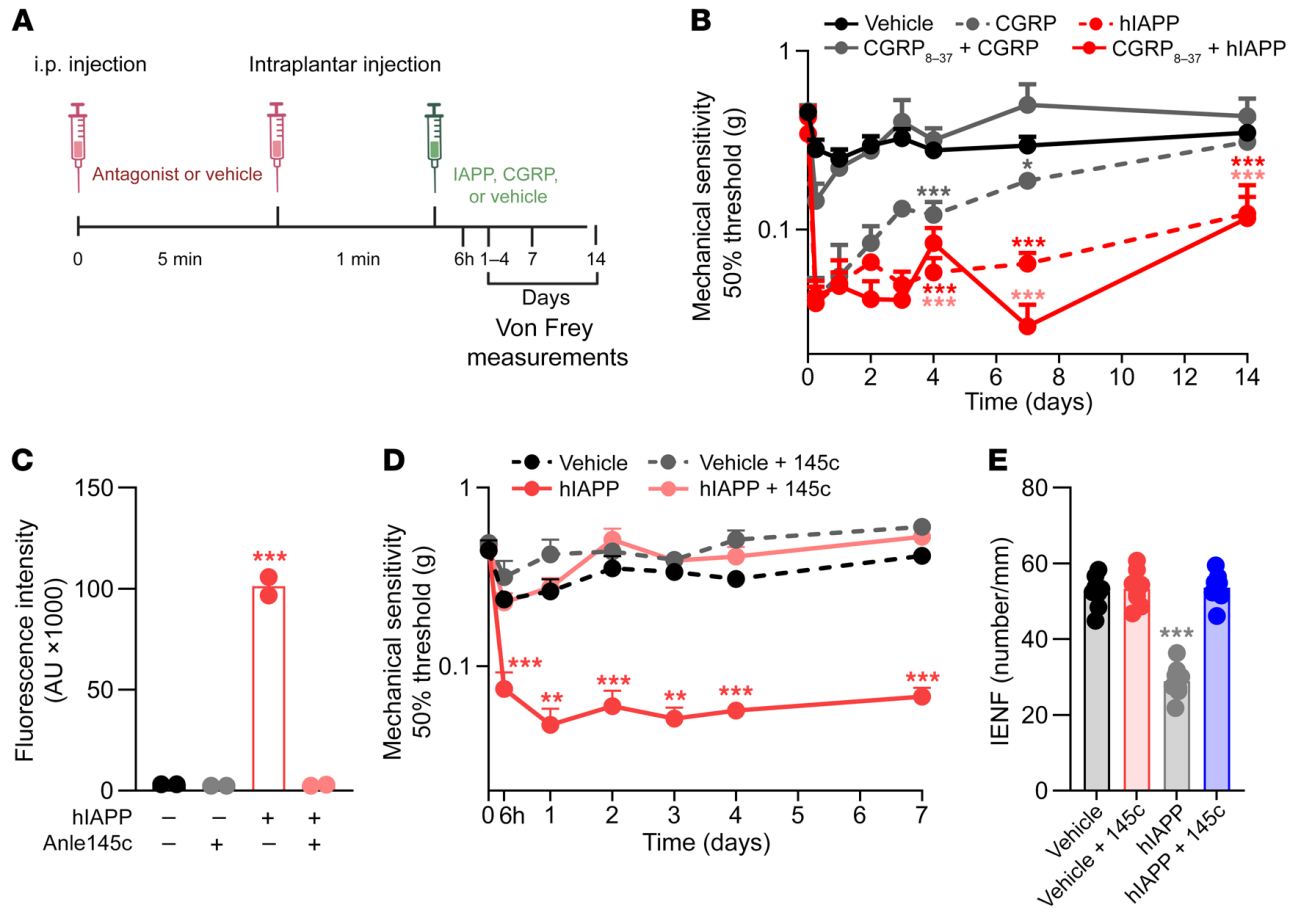
Human IAPP reduces mechanical thresholds independent of its receptors and anle145c prevents this action in WT mice. (**A**) Schematic diagram showing the timeline and administration routes for **B**. (**B**) Mechanical sensitivity after antagonist (250 μg/kg, CGRP_8–37_) injection (*n* = 13) or saline injection (*n* = 13) prior to intraplantar injection of hIAPP (1,000 fg, *n* = 8) or CGRP injection (5 μg, *n* = 5) in one paw and saline (*n* = 13) in the other paw. Both male and female mice were used. (**C**) Fluorescence of thioflavin T after 24 hours. Fluorescence intensity is determined by the concentration of amyloid fibrils. (**D** and **E**) Course of mechanical sensitivity (*n* = 8) (**D**) and plantar IENFs at 7 days (*n* = 8) (**E**) after intraplantar injection of hIAPP (1,000 fg/5 μL) with and without anle145c (1 nM), or vehicle. Treatments were prepared and incubated for 24 hours at room temperature before injection into male and female WT mice (*n* = 8). (**B**, **D**, and **E**) Two-way ANOVA with Tukey’s test; *P < 0.05, **P < 0.01, ***P < 0.001 vs. vehicle. (**C**) One-way ANOVA with Dunnett’s test; ***P < 0.001 vs. vehicle. Data are expressed as mean ± SEM.

hIAPP has 43% amino acid sequence identity with hCGRP and binds to the same IAPP/CGRP receptors (22). Therefore, hIAPP may induce effects through direct actions on IAPP/CGRP receptors. In contrast to CGRP, hIAPP is toxic to rat insulinoma cells, hippocampal neurons, and astrocytes in vitro, independent of receptors (23, 24). To evaluate whether hIAPP-induced allodynia requires IAPP/CGRP receptors, we blocked these receptors by systemic and local administration of the IAPP/CGRP receptor antagonist CGRP_8–37_ (Figure 4A). CGRP_8–37_ completely blocked the development of CGRP-induced allodynia. In contrast, the same dosing schedule of CGRP_8–37_ did not block hIAPP-induced allodynia (Figure 4B).

To test whether hIAPP aggregation is required for the development of neuropathy, we added an inhibitor of hIAPP aggregation (anle145c) (25) to hIAPP before injection. As has been observed before (25), anle145c completely inhibited hIAPP fibril formation in solution. Interestingly, anle145c completely prevented hIAPP-induced allodynia and reduction in IENF density (Figure 4, C–E).

**Figure 5:**
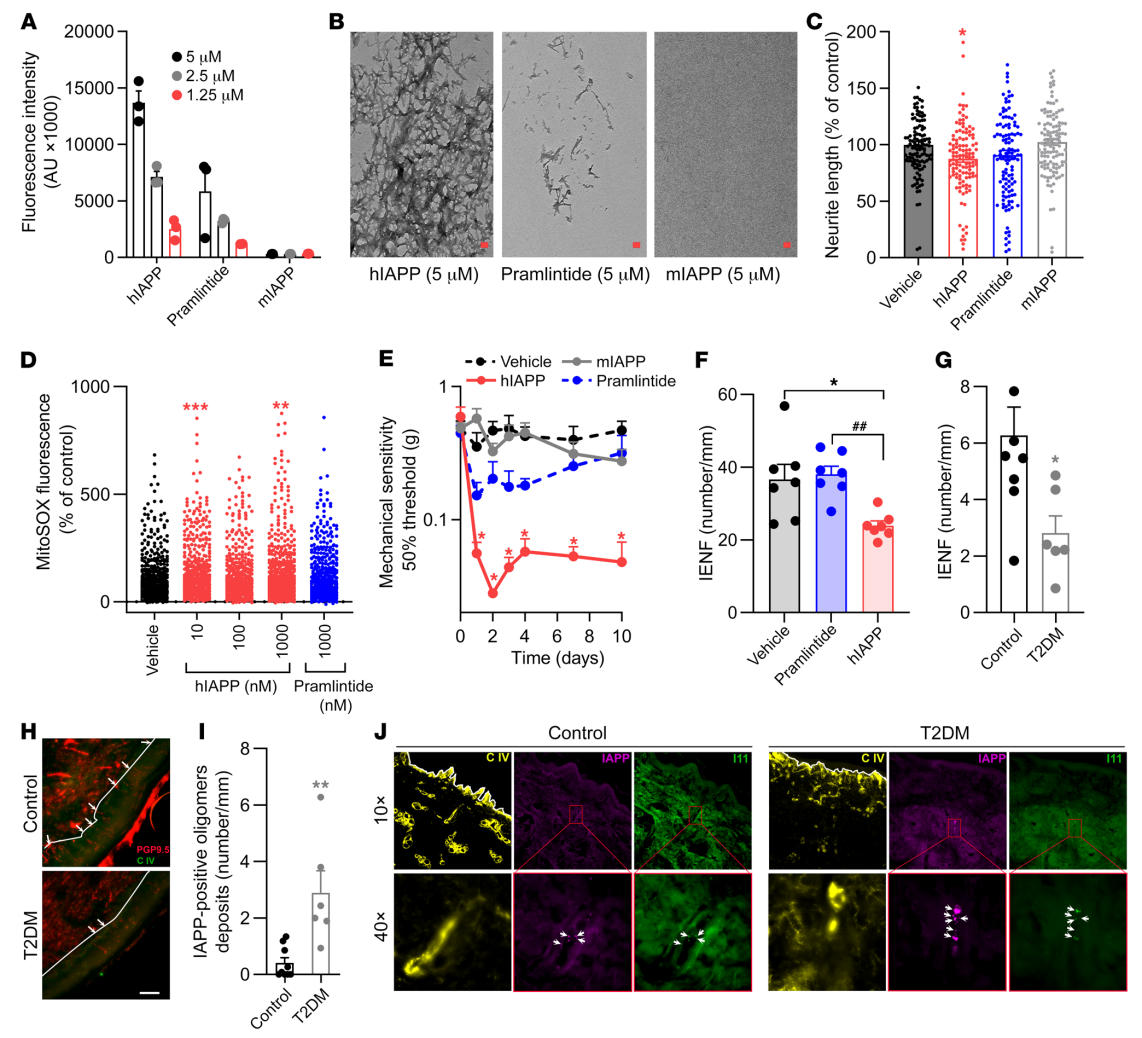
Aggregation of human IAPP is required to induce neuropathic pain. (**A** and **B**) Fluorescence (indicates amount of amyloid fibrils) of thioflavin T (**A**) and transmission electron microscopy imaging (scale bars: 0.2 nm) (**B**) after 24 hours of incubation of hIAPP, mIAPP, or pramlintide. (**C**) Sensory neurons were treated with 100 nM hIAPP, mIAPP, pramlintide, or saline for 24 hours. The average neurite length per neuron was assessed and expressed as the percentage length per neuron of vehicle-treated neurons (*n* = 8; *n* represents a DRG culture of 1 mouse). (**D**) Mitochondrial ROS level in cultured DRG neurons incubated with hIAPP or pramlintide (10, 100, and 1,000 nM). Measurements are per cell from 3 different cultures, *n* = 551–654 neurons per group. (**E** and **F**) Course of mechanical sensitivity (*n* = 5) (**E**) and IENF density (*n* = 7) on day 6 (**F**) after intraplantar injection of 1,000 fg hIAPP, mIAPP, and pramlintide in male and female WT mice. (**G**) Density of IENFs in skin of T2DM subjects (*n* = 6) and non-T2DM controls (*n* = 9). (**H**) Representative images of **G** stained for the pan-neuronal marker PGP9.5 and collagen IV (lines indicate the border between dermis and epidermis; white arrows represent the IENF; scale bar: 20 μm). (**I**) IAPP-positive oligomers in skin of T2DM subjects (*n* = 6) and non-T2DM controls (*n* = 9). (**J**) Representative images of **I** stained for collagen IV (C IV), IAPP, and oligomers (I11). IAPP- and oligomer-positive spots are indicated by arrowheads. Lines indicate the border between dermis and epidermis. (**C** and **D**) One-way ANOVA with Dunnett’s test; *P < 0.05, **P < 0.01, ***P < 0.001. (**E** and **F**) Two-way ANOVA with Tukey’s test; *P < 0.05 vs. vehicle; ^##^*P* < 0.01 vs. pramlintide. (**G** and **I**) Unpaired *t* test; *P < 0.05, **P < 0.01. Data are expressed as mean ± SEM.

To further investigate whether aggregation of IAPP is required for its neurotoxic effects, we tested mouse IAPP (non-aggregating and non-amyloidogenic) and a mutant human IAPP (pramlintide, non-amyloidogenic) in vitro. Both of these non-amyloidogenic IAPP variants activate IAPP/CGRP receptor subtypes (26). Thioflavin T fluorescence assays and transmission electron microscopy showed that mIAPP indeed did not form amyloid fibrils, whereas pramlintide only showed minor fibril formation (Figure 5, A and B). In contrast, hIAPP formed abundant amyloid fibrils (Figure 5, A and B). Treatment of cultured sensory neurons with amyloidogenic hIAPP reduced neurite outgrowth by 13% compared with vehicle control. In contrast, mIAPP or pramlintide did not affect neurite outgrowth in sensory neurons in vitro (Figure 5C).

Mitochondria are required for a wide range of cellular processes, including the regulation of neuronal functions (27). Mitochondria are the primary source of adenosine triphosphate (ATP) and reactive oxygen species (ROS). Mitochondria are responsible for neurotransmitter release, neuronal excitability, neuronal signaling, and plasticity. Increased ROS production in the peripheral and central nervous systems is associated with both inflammatory and neuropathic pain (27). To investigate the effect of hIAPP on mitochondrial function, cultured sensory neurons were treated with amyloidogenic hIAPP and non-amyloidogenic hIAPP (pramlintide). Intriguingly, amyloidogenic hIAPP induced mitochondrial superoxides in sensory neurons in culture. In contrast, pramlintide did not induce mitochondrial superoxide production in sensory neurons (Figure 5D). Next, we determined whether IAPP aggregation is required for signs of DPN in vivo. Intraplantar injection of mIAPP into WT mice did not affect mechanical thresholds (Figure 5E), while the same dose of hIAPP reduced mechanical thresholds for at least 10 days. Intraplantar injection of pramlintide slightly reduced mechanical thresholds, yet this reduction was less in magnitude and duration compared with that of hIAPP (Figure 5E). Injection of pramlintide did not affect IENF density, while the same dose of hIAPP caused a reduction of approximately 50% (Figure 5F). Overall, these data indicate that the ability of IAPP to form aggregates/fibrils is required for its neurotoxicity in vitro (28), and to induce allodynia and reduce IENF density in vivo.

hIAPP can accumulate in nervous tissues, e.g., in hippocampal neurons of hIAPP-transgenic mice and rats, and form aggregates that are associated with neurological deficits (29). hIAPP oligomers destabilize cell membranes and form membrane pores (30). Therefore, we assessed whether IAPP oligomers are present in the dorsal root ganglia (DRGs) that contain the soma of sensory neurons. Interestingly, IAPP-positive oligomers were detected in DRGs of hIAPP mice but not in DRGs of WT mice (Supplemental Figure 4). Next, we assessed the presence of hIAPP oligomers in the skin of T2DM patients with neuropathy (Supplemental Table 1). T2DM patients had neuropathy (Supplemental Table 1) and a reduction in IENF density compared with non-T2DM controls (Figure 5, G and H). Intriguingly, T2DM patients with neuropathy had significantly more IAPP-positive oligomers in the skin compared with controls (Figure 5, I and J). These data suggest that potential toxic aggregates of hIAPP are more abundant in the skin of T2DM neuropathy patients.

## Discussion

Although the cause of neuropathy in T2DM is not fully understood, three characteristics of T2DM may contribute to the development of diabetic neuropathy: hyperglycemia, obesity, and amyloidogenic IAPP (5, 12). Here, we present evidence that amyloidogenic hIAPP is a contributor to the development of diabetic neuropathy. Diabetic and non-diabetic mice expressing hIAPP developed signs of neuropathy such as allodynia and nerve damage in the skin. Importantly, the aggregation of IAPP is required to induce these signs of neuropathy. Thus, in addition to hIAPP aggregation being associated with dysfunction and death of pancreatic β cells in T2DM (6), hIAPP also damages peripheral sensory neurons and contributes to neuropathy development. Importantly, we found that hIAPP-positive oligomers are present in hIAPP mice and also in the skin of T2DM patients. These findings not only add to earlier findings that IAPP oligomers are found outside of the pancreas, they also support the notion that hIAPP is a contributor to diabetic neuropathy in humans.

Ob/Ob mice with hyperglycemia exhibit T2DM neuropathy and are often used to study T2DM neuropathy (31). Similarly, other mouse models with metabolic disturbances have been used, such as the db/db mouse (32). However, these mice are not an ideal model for human T2DM, because they lack human IAPP expression and the death of islet β cells when disease progresses. hIAPP Ob/Ob mice become insulin resistant and hyperglycemic and develop pathogenic amyloid deposits in pancreatic islets due to overproduction of hIAPP by the islet β cells (6, 14). Given the accumulating evidence that aggregating hIAPP contributes to development of T2DM (3), we propose that hIAPP Ob/Ob mice are a better model to study T2DM, diabetic neuropathy, and potentially other disease-associated symptoms, as compared with Ob/Ob and db/db mice.

Hyperglycemic Ob/Ob mice were found to be more sensitive to hIAPP-induced allodynia than WT mice. Although hyperglycemia may not be the only driver of diabetic neuropathy, it may predispose to IAPP-induced hypersensitivity because hIAPP-induced cell toxicity is exacerbated by hyperglycemia-induced glycated insulin (33). In addition, obesity sensitizes neurons to pain-inducing stimuli (34), and hyperglycemia, as well as obesity-related insulin resistance, increases IAPP transcript levels and IAPP biosynthesis in pancreatic islet β cells (35). Overall, these findings possibly explain why hIAPP Ob/Ob mice have higher mechanical sensitivity than non-obese hIAPP mice.

Our findings show that aggregation of hIAPP is necessary for the development of signs of neuropathy, suggesting that non-receptor-mediated mechanisms are probably involved. Indeed, we identified that hIAPP-induced neuropathy does not involve the IAPP/CGRP receptor. Aggregation of IAPP is required to exert its damaging effect on neurons in vitro, and blocking hIAPP aggregation, using the small molecule inhibitor anle145c (25), prevented hIAPP-induced neuropathy in vivo. Pramlintide, a non-amyloidogenic hIAPP variant, did show some fibril formation in vitro and induced some small neuropathy-like effects in vitro and in vivo in mice, but considerably less as compared with hIAPP. Since pramlintide is used in clinical practice (36), its potential neuropathic effects warrant further investigation.

In T2DM patients, small nerve fiber pathology is observed early in the development of painful DPN, sometimes even prior to the presence of hyperglycemia (37). In this study, we found more abundant hIAPP oligomers in the dermis of patients with T2DM neuropathy compared with non-diabetic controls. This raises the question of why hIAPP depositions were found in the dermis if small nerve fiber degeneration and a reduction in IENF density are related to nerve terminal loss in the epidermis. Likely, hIAPP oligomers also harm nerve fibers in the dermis, ultimately contributing to the degeneration of nerve fiber terminals in the epidermis. In line with this rationale, different neurotoxic drugs (e.g., chemotherapeutics) target neurons at different levels, but degeneration often begins in the epidermis (38). In addition, hIAPP aggregates may exist at other areas of the peripheral sensory nervous system that we are unable to evaluate with our skin biopsies. In this context, we have demonstrated the presence of hIAPP aggregates in DRGs of hIAPP mice, but not in WT mice (Supplemental Figure 4), suggesting that similar aggregates may also arise in humans.

How hIAPP mechanistically causes neuropathy remains unclear. hIAPP aggregation, with formation of oligomers and amyloid fibrils, induces cytotoxicity of pancreatic islet β cells through various mechanisms, including membrane disruption, impaired mitochondrial function, and autophagy malfunction (39). Many of these cellular abnormalities, especially mitochondrial defects (27, 40), have been implicated in pain conditions and the development/progression of diabetic neuropathy, as well as in other forms of amyloid neuropathy (12). We found increased mitochondrial ROS levels and reduced neurite outgrowth in hIAPP-treated sensory neurons, supporting the notion that aggregating hIAPP induces neurotoxicity through mitochondrial damage (12). These pathogenic mechanisms may contribute to DPN not only by impacting neurons, but also other cell types involved in neuropathy, such as macrophages, microglia, satellite glial cells, Schwann cells, and endothelial cells (12). Future studies are needed to determine which pathogenic pathways and which cell types are involved in hIAPP-mediated DPN.

In conclusion, our data show that human IAPP induces signs of peripheral neuropathy in the absence of hyperglycemia and aggravates mechanical allodynia in hyperglycemic obese (Ob/Ob) mice. Therefore, inhibition of hIAPP aggregation is a novel approach to treat and/or prevent DPN, as it plays a crucial role in the progression of T2DM-associated neuropathy.

## Methods

### Animals.

Power calculations were performed to estimate the sample size needed to detect a minimal predefined effect size. All animals were randomly allocated to a group before the start of any measurements or treatment. Experiments were conducted using both male and female mice aged 8–16 weeks. Observers performing behavioral experiments were blinded with respect to treatment/genotype groups. Mice were housed in groups under a 12-hour light/12-hour dark regime, and food and water were available ad libitum. The cages contained environmental enrichments including tissue papers and shelter opportunities. Mice were acclimatized to the experimental setup for 1–2 weeks prior to the start of each experiment. To avoid potential cage bias, animals were randomly assigned to the different groups before the start of experiments, treatment groups were equally divided per housing cage, and experimenters were blinded to the treatments and genotypes.

Four groups of mice were used: (a) transgenic mouse model of T2DM (obese [Ob/Ob] mice that produce hIAPP in their pancreatic islet β cells; hIAPP Ob/Ob), (b) non-obese hIAPP-transgenic mice, (c) Ob/Ob mice, and (d) WT mice.

The generation of these 4 separate sublines from the original GG2653 line (hIAPP × Leptin Ob, on a C57BL/6 background) was previously described (14, 41). The generation of the mice was performed in 2 breedings: (a) Ob heterozygous (Ob/+) × Ob heterozygous (Ob/+) and (b) hIAPP homozygous/Ob heterozygous × hIAPP homozygous/Ob heterozygous.

Ob/+ mice were mated with Ob/+ mice to generate non-transgenic Ob/Ob mice (homozygotes) and WT mice. To maintain this strain, WT mice (+/+) were mated with Ob/+ mice.

hIAPP-homozygous Ob/Ob mice and hIAPP-homozygous non-obese mice were generated by mating of hIAPP-homozygous Ob/+ mice with hIAPP-homozygous Ob/+ mice. The distinction between Ob/Ob, Ob/+, and WT littermates in both breedings was performed by genotyping of a leptin-Ob gene PCR product of 250 bp using the restriction enzyme DdeI. This results in 2 DNA fragments for WT mice (150 bp and 100 bp), while the Ob point mutation creates an additional DdeI site within the 100 bp fragment, resulting in 3 DNA fragments after DdeI digestion for Ob/+ mice and complete loss of the 100 bp fragment for Ob/Ob mice.

### Glucose, IAPP, and insulin measurements.

Blood was obtained by cheek puncture from nonfasted mice at age 10 weeks and at the end of the experiments (at an age between 15 and 18 weeks). Blood was collected in EDTA tubes (MiniCollect, Greiner) and saved on ice until centrifugation at 900*g* for 5 minutes at 4°C. Plasma was taken, divided into aliquots, and stored at –80°C until analysis.

Glucose was measured in EDTA plasma using the hexokinase method.

Insulin was measured in EDTA plasma using a radioimmunoassay (RI-13K, Merck Millipore). IAPP was measured by ELISA (EZHA-52K, Merck Millipore).

### Drug dilution and administration.

hIAPP, mIAPP (both obtained from Bachem), and pramlintide (obtained from AnaSpec) were dissolved in dH_2_O to obtain stock solutions of 200 μg/mL; aliquots were stored at –80°C. Human calcitonin gene–related peptide α (CGRPα) and human calcitonin gene–related peptide 8–37 (CGRP_8–37_) were obtained from Bachem and dissolved in 0.9% NaCl (1 mg/mL) before injection. For behavioral experiments, WT male mice received i.v. injection of hIAPP (40 μg/kg, 4 μg/kg) or vehicle (0.9 % NaCl) at an age between 8 and 16 weeks.

The i.v. injection dose was calculated based on the estimation that WT mice have 2 mL of blood and the physiological range of plasma IAPP concentrations in healthy WT mice is 20–100 pM (28). Thus, i.v. injection of 40 μg/kg would correspond to 125 nM in blood, which is approximately 1,000 times higher than the physiological range.

hIAPP (10–1,000 fg/5 μL), mIAPP (1,000 fg/5 μL), or pramlintide (1,000 fg/5 μL) was injected intraplantarly in one paw. The other paw received the same volume of 0.9 % NaCl. For intraperitoneal injections, mice received the IAPP/CGRP antagonist (CGRP_8–37_, 250 μg/kg) or vehicle (0.9% NaCl) at 5 minutes before the intraplantar injection of hIAPP. Also at 1 minute before intraplantar injection of hIAPP (1,000 fg/5 μL) or CGRP (5 μg/5 μL), mice received an intraplantar injection of CGRP_8–37_ (5 μg/5 μL).

For intraepidermal nerve fiber (IENF) quantification, WT mice received an intraplantar injection of 1,000 fg hIAPP or pramlintide in 5 μL saline or vehicle. For blocking hIAPP aggregation with anle145c, monomeric hIAPP was dissolved with hexafluoroisopropanol (HFIP) to obtain a monomeric stock solution. Anle145c was dissolved as 10 mM stock solution in DMSO. A 50 μM hIAPP solution was prepared from this stock in Tris 10 mM, NaCl 100 mM, pH 7.4, and incubated overnight at room temperature with or without a 20-fold molar excess of anle145c (1 mM) with 10% DMSO. This solution of hIAPP was diluted to 50 pM in saline before intraplantar administration of 5 μL to the mice.

### IENF quantification.

Skin of hind paw was taken from mice after euthanasia, then incubated with Zamboni’s fixative overnight at 4°C, rinsed overnight in 30% sucrose in PBS at 4°C, and then cryoembedded in mounting media (OCT compound). The skin was cryosectioned at 20 μm for immunohistochemistry; sections were incubated with rabbit anti-PGP9.5 antibody (1:1,000; Sigma-Aldrich) and goat anti–collagen IV antibody (1:200; Southern Biotech) for 24 hours at 4°C. Sections were then rinsed 3 times in PBS plus 0.3% Triton X-100 and incubated with Alexa Fluor 594–labeled donkey anti-rabbit antibody (1:500; Life Technologies) or Alexa Fluor 488–labeled donkey anti-goat antibody (1:500; Life Technologies) for 2 hours in the dark at room temperature, and with DAPI (1:5,000) for 5 minutes, before being rinsed (2 times in distilled water) and mounted onto slides. A stack of 12 images per hind paw skin was obtained using a Zeiss confocal microscope (×40 objective), and the number of nerve fibers that crossed from the dermis to the epidermis per linear millimeter of skin was quantified.

### Real-time quantitative PCR.

Total RNA was isolated from hind paw skin that was isolated on day 6 after intraplantar injection of vehicle, or of 1,000 fg/5 μL hIAPP or pramlintide, using TRIzol (Invitrogen) and RNeasy mini kit (Qiagen) according to the manufacturer’s protocol.

 cDNA was synthesized using iScript Reverse Transcription Supermix, in accordance with the manufacturer’s instructions (Bio-Rad). Real-time quantitative PCR was then performed with iQ SYBR Green Supermix (Invitrogen). We used an amount of cDNA corresponding to 1–5 ng of RNA input per quantitative PCR reaction and the primer pairs shown in Table 1. mRNA expression is represented as relative expression = 2^Ct(average of housekeeping genes 18S, HPRT, β-actin) – Ct(target)^.

### Pain measurements.

Mechanical sensitivity was measured by Von Frey hairs (Stoelting), and the 50% paw withdrawal threshold was calculated using the up-and-down method. Briefly, Von Frey filaments were placed to the plantar surface of the paw for a maximum of 5 seconds. In the event of a nonresponse after the first filament (0.4 g), the next filament with a higher force was applied. In the event of a response, the filament with the lower force was used. A minimum of 30 seconds was taken between the application of filaments. After the first change of direction, 4 readings were taken (42). Thermal sensitivity was assessed by Hargreaves test (IITC Life Science). In short, the Hargreaves test was conducted in a Perspex enclosure (box) with a heated (32°C) glass bottom. Underneath the animals, a radiant heat source was focused at the plantar surface of the hind paw. The time taken until a withdrawal response from the heat stimulus was recorded as the withdrawal latency. Paws were measured at least 3 times with at least 30 seconds between measurements. In WT C57BL/6 mice, the intensity of the light source was tuned to generate withdrawal latencies of 8–10 seconds, with a 20-second cutoff to avoid tissue injury (43).

Spontaneous pain was measured by the conditioned place preference test (CPP; Stoelting), as previously described (44, 45). In short, a 3-chamber box (A, B, and C) was used (chambers A and C measure 18 × 20 cm), with the B chamber being a smaller chamber connecting A and C divided from the neighboring chambers by a divider with an entrance. The A and C chambers had various patterned walls and floors that were texturally different. At day 1 (preconditioning) animals were allowed to move freely for 30 minutes, and the time spent in each chamber was recorded. At days 2–4, the mice were conditioned with daily intraperitoneal injections of vehicle or the painkiller gabapentin (100 mg/kg; Sigma-Aldrich). In the morning, the animal was placed for 30 minutes in 1 closed chamber (black room), 1 minute after receiving vehicle. In the afternoon (3 hours later), the mouse was placed for 30 minutes in the other chamber (white room) 1 minute after receiving gabapentin treatment. At day 5 after conditioning, the animals were placed in the start chamber (chamber B) and allowed free access for 30 minutes to either chamber (A or C). The time spent in the chambers was recorded. To define treatment effect, the mean of the time spent in the treatment-associated chamber (white room) during adaptation (day 1; preconditioning) was subtracted from the time spent in that chamber on the test day (day 5; postconditioning). A significantly longer stay in the white room after conditioning indicates the presence of spontaneous pain.

### Primary cell cultures.

Adult mouse dorsal root ganglia (DRGs) were dissected after mice were killed and then placed in ice-cold dissection medium (HBSS without Ca^2+^ and Mg^2+^ [Gibco 14170-088], 5 mM HEPES [Gibco 15630-049], and 10 mM glucose [Sigma-Aldrich G8769]). After dissection, axons were cut off and subsequently the DRGs were digested, in order to get a cell suspension, with a mixture of HBSS without Ca^2+^ and Mg^2+^, 5 mM HEPES, 10 mM glucose, 5 mg/mL collagenase type XI (Sigma-Aldrich), and 10 mg/mL Dispase (Gibco), for 30–40 minutes at 37°C in a 5% CO_2_ incubator. After that, DMEM (31966-021, Gibco) with 10% heat-inactivated FBS (F9665, Sigma-Aldrich) was added to inactivate the enzyme mixture. Cells were centrifuged for 5 minutes at 79*g*, resuspended in DMEM (Gibco) containing 10% FBS (Gibco), 2 mmol/L-glutamine (Gibco), and 10,000 IU/mL penicillin-streptomycin (Gibco), and plated on glass coverslips coated with poly-l-lysine (0.01 mg/mL; Sigma-Aldrich) and laminin (0.02 mg/mL; Sigma-Aldrich) in a 5% CO_2_ incubator at 37°C for 1 day. To investigate the effect of hIAPP on neurite outgrowth, cells were incubated with various concentrations of hIAPP (0.1–1,000 nM) for 24 hours, and to investigate the effects of IAPP aggregation, 100 nM hIAPP was compared with 100 nM mIAPP, 100 nM pramlintide, or vehicle for 24 hours.

### Neurite outgrowth analysis.

After incubation with hIAPP, mIAPP, pramlintide, or vehicle, DRG cells were fixed with 4% paraformaldehyde for 10 minutes at room temperature and washed 3 times with PBS. DRG cells were then permeabilized with PBS with 0.05% Tween (PBST) 3 times for 5 minutes at room temperature, incubated in blocking solution (5% normal goat serum and 1% BSA in PBST) for 30 minutes at room temperature, and then incubated overnight at 4°C with rabbit anti–β_3_-tubulin antibody (1:1,500; Abcam, ab18207) and mouse anti-NeuN antibody (1:500; MAB377, Sigma-Aldrich). The following day, the cells were washed 3 times in PBST, incubated with Alexa Fluor 488–labeled donkey anti-rabbit antibody (1:1,000; Life Technologies) and Alexa Fluor 568–labeled donkey anti-mouse antibody (1:1,000; Life Technologies) in the dark for 1 hour, washed twice with PBST for 5 minutes and once with PBS for 5 minutes, incubated with DAPI (1:5,000) for 5 minutes at room temperature, washed twice for 5 minutes with distilled water, and then mounted onto slides. Confocal images were acquired with a Hamamatsu Camera C13440 on an Olympus IX83 microscope (Olympus Life Sciences) at ×20; 5 images randomly were taken from each well of each treatment (3 wells per condition). ImageJ (NIH) with Neuralmetrics macro plugin (46) was used to trace the neurons and their neurites automatically and measure the total neurite length. The average neurite length per neuron was calculated, and all values were expressed as percentage of the vehicle control.

### Mitochondrial ROS.

Cultured DRG neurons were incubated with 200 nM MitoTracker Deep Red (M22426, Invitrogen) and 5 μM MitoSOX Red (M36008, Invitrogen), an indicator of mitochondrial superoxide, for 20 minutes, protected from light exposure. After washing, 4 images were taken of random areas of each well, with, in total, 2 wells per condition in each culture of DRG from 1 mouse. In total, 3 cultures were performed from 1 female and 2 male mice. Pictures were taken with an Olympus IX83 fluorescence microscope. Bright-field images were used to distinguish neurons from other cell types and select them for analysis with ImageJ software.

### Thioflavin T fluorescent assay.

Thioflavin T (ThT) assay was conducted in a standard 96-well black microtiter plate using a plate reader (CLARIOstar plus, BMG LABTECH). Per well, 20 μL of each peptide (hIAPP, mIAPP, or pramlintide) was added to 180 μL buffer. The final buffer concentrations were 10 μM ThT, 100 mM NaCl, 10 mM Tris (pH 7.4). For the aggregation inhibitor experiment, per well, 20 μL of hIAPP, anle145c, hIAPP plus anle145c, or vehicle was added to 180 μL buffer. The final buffer concentrations were 20 μM ThT, 10 mM Tris, 150 mM NaCl, 2.5% DMSO (pH 7.4). The final IAPP and anle145c concentrations were 12.5 μM and 250 μM, respectively.

The plate was covered using a clear Viewseal sealer (Greiner) to prevent evaporation during the experiment. The plate was shaken at 500 rpm for 30 seconds immediately prior to the first measurement. To determine the formation of fibrils, the fluorescence intensity was followed in time. Binding of ThT to amyloid fibrils results in an increase in fluorescence (47).

The fluorescence was measured every 5 minutes (until 24 hours) at room temperature from the top of the plate with excitation at 435 nm and emission at 530 nm. Measurements were performed in triplicate for each condition/concentration.

### Transmission electron microscopy.

Aliquots (4.20 μL) of 5 μM of hIAPP, mIAPP, and pramlintide were blotted on carbon-coated 200 mesh copper grids, glow-discharged for 2 minutes. Then the samples were negatively stained with 4% uranyl acetate 2 times for 1 minute. The grids were dried and examined using an FEI-CM 120 transmission electron microscope (Thermo Fisher Scientific) equipped with a Gatan US1900 charge-coupled device (CCD) camera.

### IAPP and oligomer staining.

Lumbar DRGs (L3–5) from WT and hIAPP mice were collected between the ages of 14 and 18 weeks, fixed in 4% paraformaldehyde, embedded in OCT compound (Sakura), and frozen at –80°C.

For immunofluorescence, cryosections (10 μm) of DRG were stained with primary mouse anti–human IAPP (1:500; ab115766 mouse monoclonal, Abcam) and rabbit anti–I11 oligomer (1:500; received from Rakez Kayed, University of Texas Medical Branch, Department of Neurology, Galveston, Texas, USA) antibodies overnight at 4°C, followed by 2 hours of incubation with Alexa Fluor 568–labeled donkey anti-mouse (1:500; Life Technologies) or Alexa Fluor 488–labeled donkey anti-rabbit (1:500; Life Technologies) fluorescent secondary antibody. Images of immunostaining were captured at ×20 using a Hamamatsu Camera C13440 on an Olympus IX83 microscope (Olympus Life Sciences).

For skin staining, samples were collected from hands or feet of T2DM subjects (*n* = 6) and non-T2DM controls (*n* = 9) undergoing surgery. Tissues were fixed in 4% paraformaldehyde overnight, then embedded in OCT compound (Sakura) and frozen at −80°C.

For immunofluorescence, cryosections (50 μm) of skin were stained with primary mouse anti–human IAPP (1:500; Ab115766 mouse monoclonal, Abcam), rabbit anti–I11 oligomer (1:500; received from Rakez Kayed), goat anti–collagen IV (1:200; Southern Biotech), and mouse anti–human protein gene product 9.5 (PGP9.5) (1:500; MCA4750GA mouse monoclonal, Bio-Rad) antibodies for 36 hours at 4°C, followed by overnight incubation with Alexa Fluor 568–labeled donkey anti-mouse (1:500; Life Technologies), Alexa Fluor 488–labeled donkey anti-rabbit (1:500; Life Technologies), or Alexa Fluor 647–labeled donkey anti-goat (1:500; Life Technologies) fluorescent secondary antibody. Nuclei were stained with DAPI (1:5,000) for 5 minutes. Immunostaining images were captured with a Hamamatsu Camera C13440 on an Olympus IX83 microscope (Olympus Life Sciences) at ×10 and ×40 magnification in *Z*-stacks using identical exposure times for all slides. Pictures were analyzed manually for double-positive (IAPP and oligomer) spots. The total length of analyzed skin was determined in order to determine the average number of IAPP-positive oligomer spots per millimeter of skin. Individuals performing this analysis were blinded to patient groups.

### Statistics.

Data are expressed as mean ± SEM and were analyzed with GraphPad Prism version 8.3 using unpaired 2-tailed *t* tests, 1-way or 2-way ANOVA, or 2-way repeated-measures ANOVA, as appropriate, followed by post hoc analysis. The post hoc analyses used are indicated for each figure. A *P* value less than 0.05 was considered to indicate statistically significant differences between treatment groups/conditions. Plasma IAPP and insulin data were analyzed after log transformation. The overall value of the area under the curve was expressed as positive by the formula –SUM(day 0 until day 4).

### Study approval.

All animal experiments were performed in accordance with international guidelines and with previous approval from the local experimental animal committee of the University Medical Center (DEC, Dierexperimentencommissie Utrecht), the national Central Authority for Scientific Procedures on Animals (CCD, Netherlands; license no. AVD115002015323), and the local experimental animal welfare body IVD (Instantie voor Dierenwelzijn Utrecht).

Human skin samples were obtained with permission from the Toetsingscommissie Biobanken UMC Utrecht committee for usage of material from the UMC Utrecht Biobank, under license TCBio-19.705.

## Author contributions

MMHA, CEH, JWMH, and NE designed the research and wrote, reviewed, and edited the paper. MMHA, BOWE, JP, and SV performed the experiments. EMB and JHC provided human skin tissues. MMHA, JP, and SV analyzed the data. JWMH and NE supervised the work.

## Supplementary Material

Supplemental data

## Figures and Tables

**Table 1:**
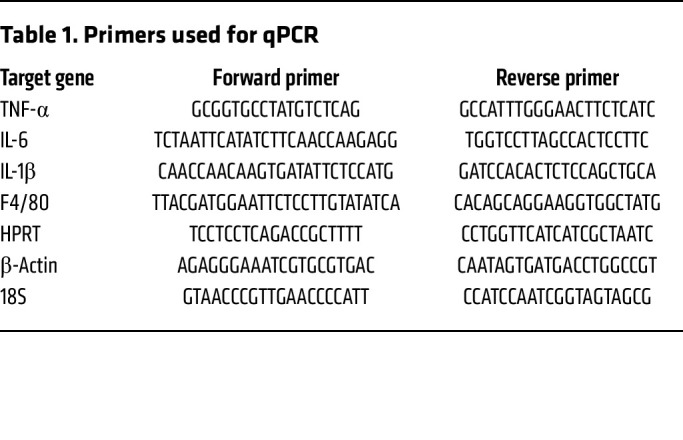
Primers used for qPCR

## Version 1 03/14/2023

In-Press Preview

## Version 2 04/17/2023

Electronic publication
