# Supplementary material for: Human IAPP is a contributor to painful diabetic peripheral neuropathy
Source: J Clin Invest. 2023 Apr 17;133(8):e156993. doi: 10.1172/JCI156993 (PMC10104883; doi:10.1172/JCI156993)
Supplement: Supplemental data [file jci-133-156993-s063.pdf]

Supplemental Data (Online Supplemental Material)

Table 1. Clinical characteristics of T2DM patients and non-T2DM controls.

**Characteristic**	**Controls**<br><br>**(n=9)**	**T2DM**<br><br>**(n=6)**
**Median age (range) years**	47 (24-67)	59 (58-72)
**Gender (n).**	Female (n=7)<br><br>Male (n=2)	Female (n=4)<br><br>Male (n=2)
**Organ**	Hand and Foot	Foot
**Tissue location**	Plantar and Dorsal	Plantar and Dorsal
**Neuropathy**	No	Yes

Abbreviations:
T2DM= type 2 diabetes mellitus

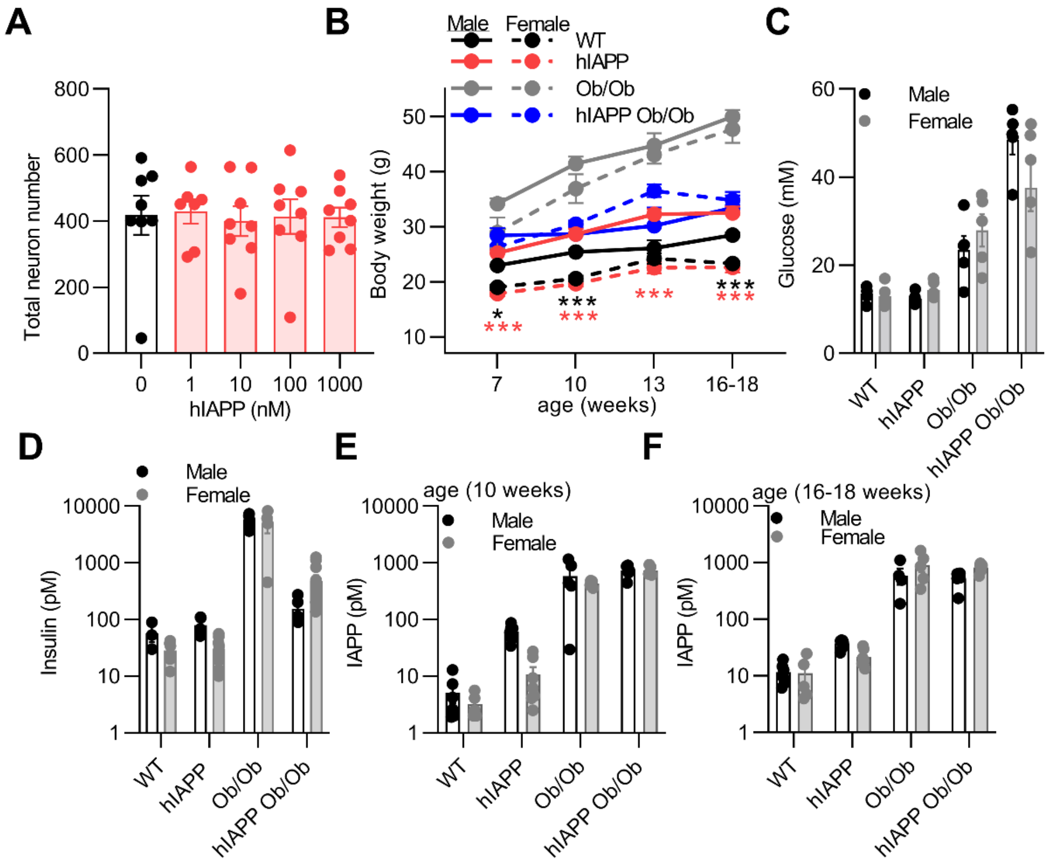

Supplementary figure 1. Human IAPP incubation with sensory neurons and metabolic parameters in male and female WT, hIAPP, Ob/Ob and hIAPP Ob/Ob mice. (**A**) Sensory neurons were cultured and treated for 24h with different concentrations of hIAPP (1-1000nM) or vehicle. The total neuron number was assessed (n=8; n represents a DRG culture of one mouse); **)** One-way ANOVA with Tukey's test. (**B**) Body weight of male mice (WT (n=6), hIAPP (n=15), Ob/Ob (n=16), hIAPP Ob/Ob (n=8)) and female mice (WT (n=5), hIAPP (n=8), Ob/Ob (n=5), hIAPP Ob/Ob (n=6); Two-way ANOVA with Tukey's test; *p < 0.05, **p < 0.01,***p < 0.001 male vs female. (**C**) Non-fasting plasma glucose level; (**D**) Non-fasting plasma insulin levels of male and female mice (**C/D;** mice age: 16-18 weeks; n=5 and, (**E, F**) Non-fasting plasma IAPP levels of male mice and female mice; n=5.(**C-F**) Two-way ANOVA with Sidak's test. Data are presented as mean ± SEM.

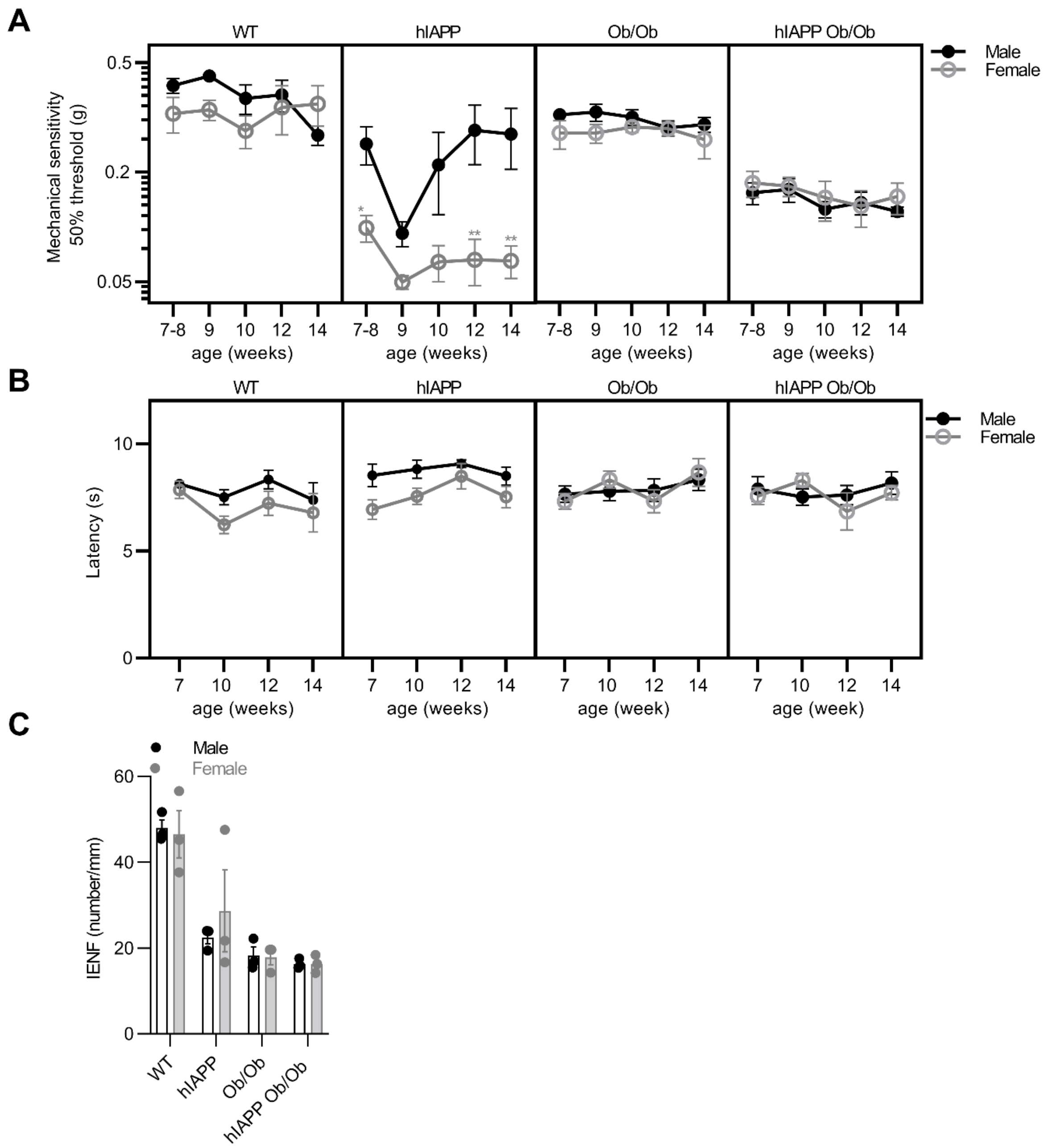

Supplementary figure 2. Pain behaviour measurements in male and female WT, hIAPP, Ob/Ob and hIAPP Ob/Ob mice. (**A**) Mechanical threshold of male mice (WT (n=6), hIAPP (n=15), Ob/Ob (n=16), hIAPP Ob/Ob (n=8)) and female mice (WT (n=5), hIAPP (n=8), Ob/Ob (n=5), hIAPP Ob/Ob (n=6)), Two-way ANOVA with Sidak's test; $*p < 0.05$, $**p < 0.01$ male vs female. (**B**) Thermal sensitivity of male mice (WT (n=5), hIAPP (n=7), Ob/Ob (n= 5) hIAPP Ob/Ob (n=7)) mice and female mice (WT (n=5), hIAPP (n=8), Ob/Ob (n=5), hIAPP Ob/Ob (n=5)); Two-way ANOVA with Sidak's test. (**C**) Quantification of IENF of the hind paw of male and female mice at 16-18 weeks of age; n=3 for each group; Two-way ANOVA with Sidak's test. Data are presented as mean ± SEM.

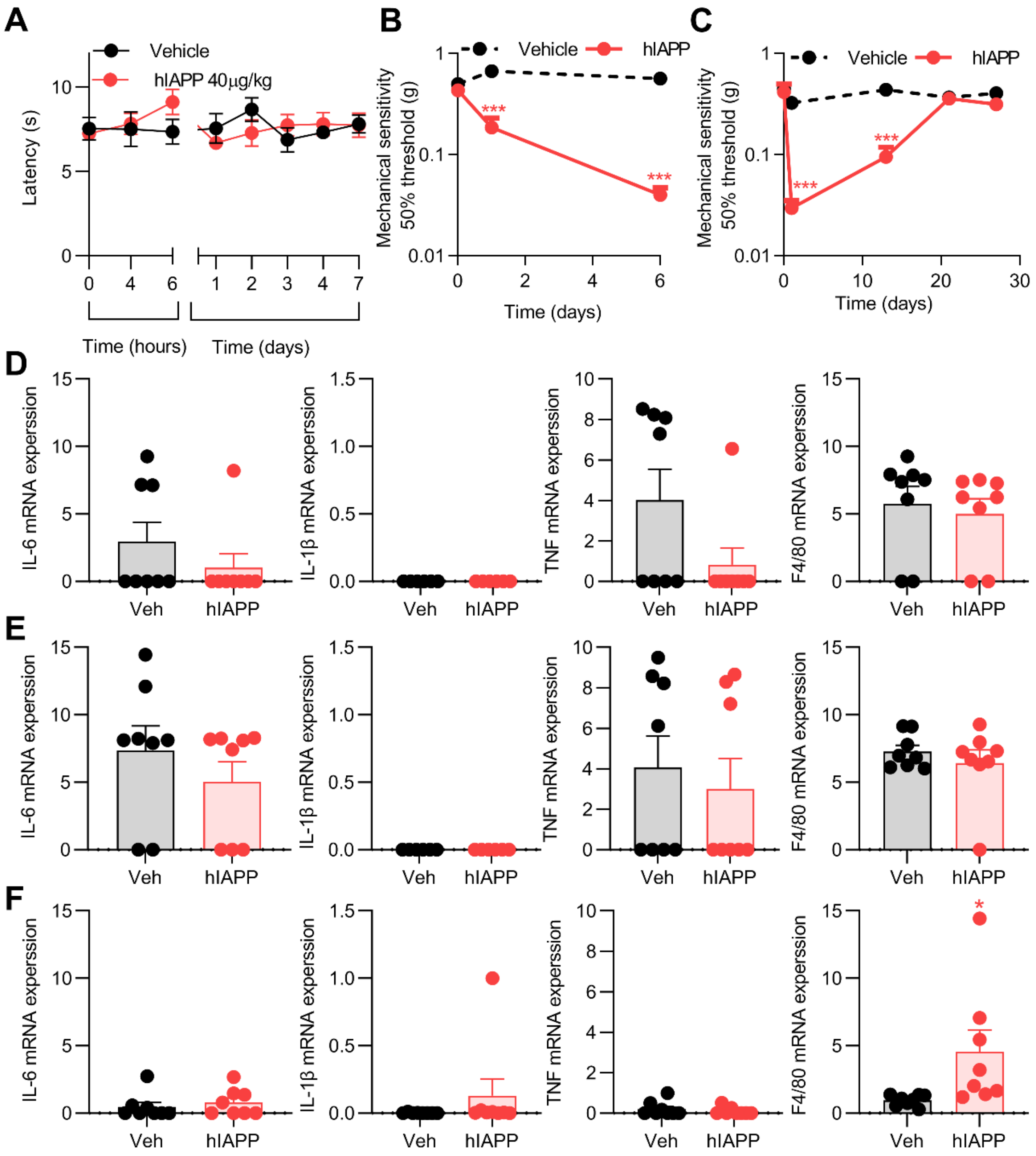

Supplementary figure 3. Human IAPP injection in the hindpaw (intraplantar) of WT mice did not induce thermal hypersensitivity or trigger expression of inflammatory cytokines. (**A**) Thermal sensitivity measured of the hind paw after intravenous injection of hIAPP (40 µg/kg n=6) or saline (NaCl (n=6)) into male WT mice; Two-way ANOVA followed by Sidak's multiple comparison test. (**B,C**) Mechanical sensitivity of the hind paw after intraplantar injection of hIAPP (1000fg, n=8) or saline (n=8) into male and female WT mice; Two-way ANOVA followed by Sidak's multiple comparison test; ***P < 0.001. (**D-F**) IL-6, IL-1β, TNF and F4/80 mRNA expression in hindpaw skin at 6 hours (**D**), 24 hours (**E**) and 6 days (**F**) after intraplantar injection of 1000 fg hIAPP or saline into WT male and female mice. Expression is normalised against housekeeping genes (average of actin, HPRT and 18S); Unpaired t test; *p<0.05, n=8. Data are presented as mean ± SEM.

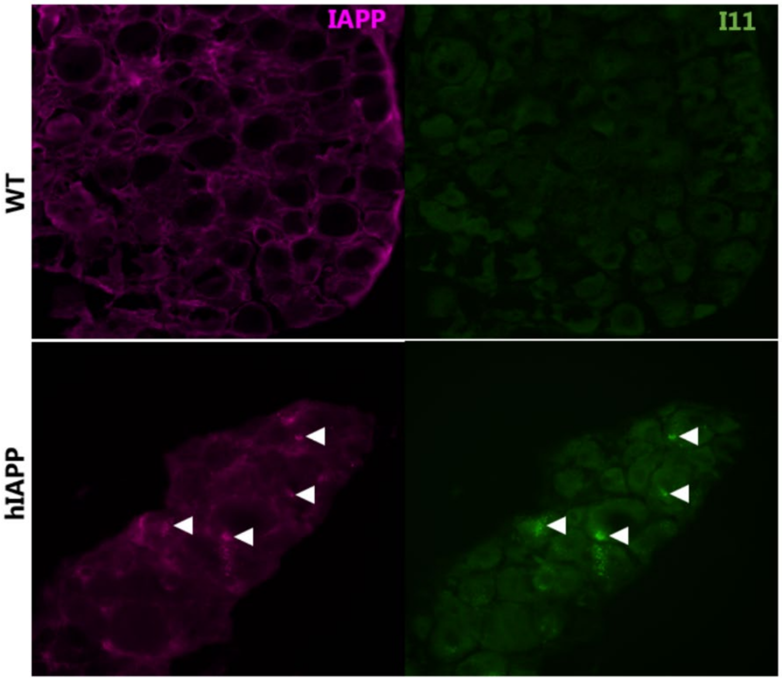

Supplementary figure 4. hIAPP oligomers present in hIAPP transgenic mice.
Representative images of the IAPP and oligomer staining (I11) in DRG of WT mice (controls) and hIAPP mice, IAPP and oligomer –positive spots are indicated by the arrowheads.
